# The cross-sectional area ratio of a specific part of the flexor pollicis longus tendon- a stable sonographic measurement for trigger thumb: a cross-sectional trial

**DOI:** 10.1186/s12891-023-06316-x

**Published:** 2023-03-27

**Authors:** Wenbin Zhu, Huan Zhou, Zhe Hu, Hongyan Chen, Juan Liu, Jin Li, Xiaoyuan Feng, Xueqin Li

**Affiliations:** 1 Ultrasonography department, Wuhan Fourth Hospital, Puai Hospital, Tongji Medical College, Huazhong University of Science and Technology, Wuhan, 430030 China; 2 The Children’s Heart Center, Wuhan Children’s Hospital (Wuhan Maternal and Child Healthcare Hospital), Tongji Medical College, Huazhong University of Science and Technology, Wuhan, 430030 China

**Keywords:** High Frequency Ultrasound, Flexor Pollicis Longus Tendon, Trigger Finger

## Abstract

**Background:**

Trigger thumb is a pathologic condition of the digital pulleys and flexor tendons. To find a cutoff value of the cross-sectional area ratio of specific parts of the flexor pollicis longus tendon to diagnosis trigger thumb in the high-frequency ultrasound examination.

**Methods:**

We evaluated 271 healthy volunteers and 57 patients with clinical diagnosis of trigger thumb. The cross-sectional area of the metacarpophalangeal joint of flexor pollicis longus tendon (C1) and the cross-sectional area of the midpoint of the first metacarpal of flexor pollicis longus tendon (C2) were analyzed.

**Results:**

There is no difference between gender, age and left and right hands in the ratio of C1 to C2 (C1/ C2). The mean of C1/ C2 in the healthy thumb was 0.983 ± 0.103, which was significantly smaller in comparison to the diseased thumb (*P* < 0.05). Based on the receiver operating characteristic curve, we chose the diagnostic cut-off value for the C1/ C2 to be 1.362 and 1.153 in order to differ a trigger thumb from children and adults.

**Conclusions:**

The C1/ C2 of the healthy thumb was relatively stable, with a mean value of 0.983 ± 0.103. The cutoff value of C1/C2 to distinguish healthy thumb from diseased thumb in children and adults were 1.362 and 1.153, respectively.

## Background

Trigger finger/ thumb is a pathologic condition of the digital pulleys and flexor tendons, with lifetime occurrence rates of 2.6% in healthy individuals and 10% in diabetics [1]. Two peaks in incidence occur: the first under the age of eight and the second (more common) in the fourth and fifth decades of life. When children get trigger finger/ thumb, it affects boys and girls equally and is most common in the thumb. In adults, women are much more likely to be affected by trigger finger, and typically, in their dominant hand [2]. The diagnosis is based on a characteristic clinical history and physical examination, such as flexion and extension disorders and tenderness on local palpation [3]. As an inexpensive and largely available imaging method, ultrasound can dynamically evaluate the surface structure of the hand and compare it with adjacent fingers and healthy contralateral [4].

The ultrasound findings of trigger finger/ thumb are as follows; a global or nodular hypoechoic thickening of the involved first annular (A1) pulley [5], increased thickness of flexor tendons, cysts, diffuse thickening of the synovial sheath, irregular internal echotexture, and tendon laceration [6]. Recently, Spirig et al. defined a cut-off value of the pulley thickness based on a simple-to-use value of 0.62 mm in order to distinguish between healthy and diseased A1 pulley [7]. In children with trigger thumb, no definite ultrasound abnormality of the A1 pulley has been found [8]. At the same time, it is difficult for us to check the A1 pulley with the 5–11 MHz transducer that we normally use [9]. Therefore, a more easily observed ultrasound parameter is needed to evaluate trigger thumb in children and adults.

To our knowledge, pediatric trigger thumb presents with focal flexor tendon enlargement at the level of the A1 pulley [8]. The thickness of flexor tendon under the A1 pulley is related to the severity of trigger thumb [10]. Our team found that the cross-sectional area of flexor pollicis longus (FPL) tendon at the metacarpophalangeal (MCP) joint (C1) and at the midpoint of the first metacarpal bone (C2), and the ratio of C1 and C2 (C1/C2) can reflect the pathological changes of trigger thumb in children and adults [11]. Therefore, in this study, we further expanded the study sample to explore the diagnostic role of specific sectional area of FPL tendon in trigger thumb.

## Methods

### Study population and clinical information

**Fig. 1:**
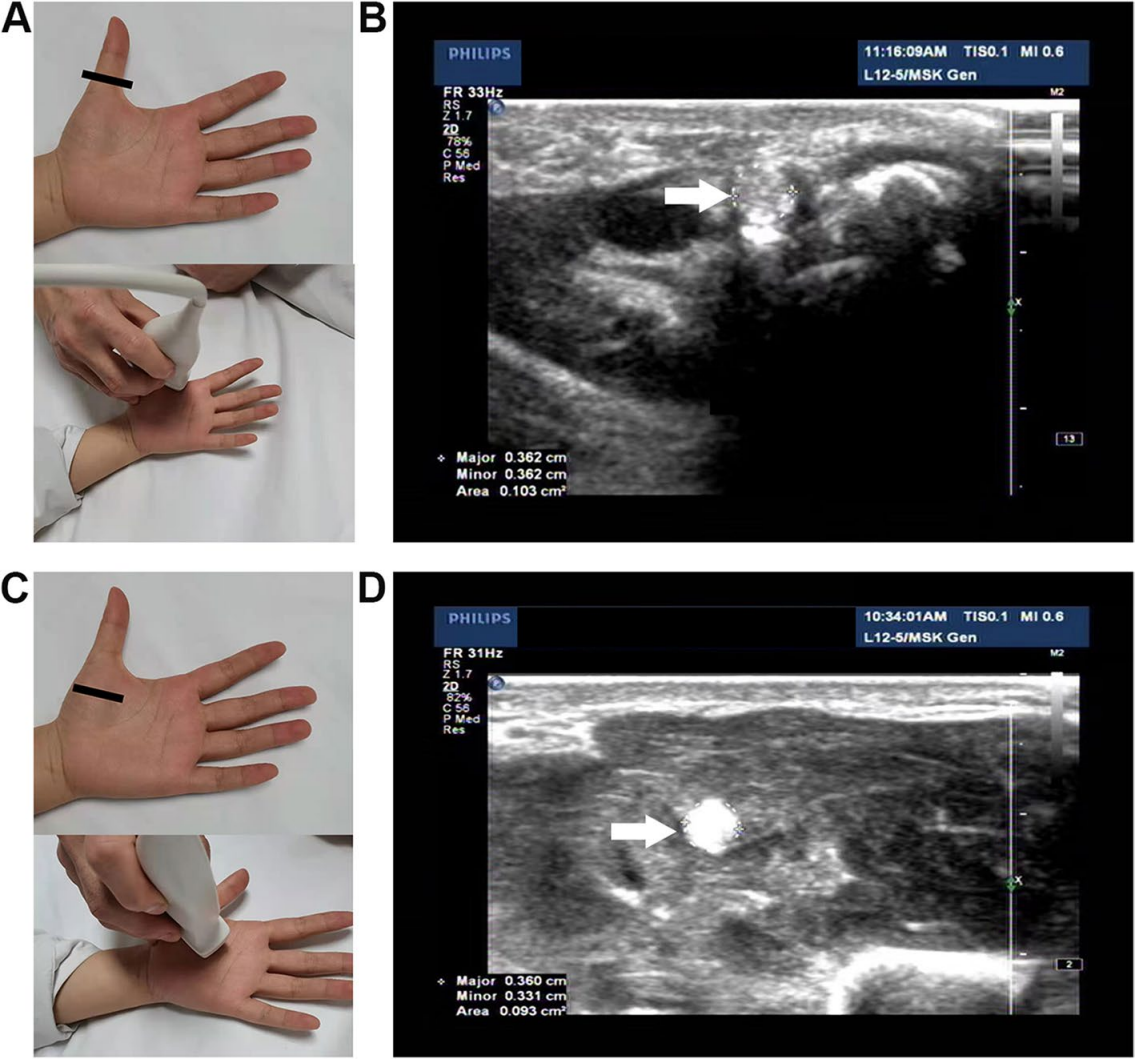
**A-B** Ultrasonic probe position (black line) and ultrasonic measurement image (dotted line) of the cross-sectional area (C1) of FPL tendon (white arrow) at the MCP joint. **C-D** Ultrasonic probe position (black line) and ultrasonic measurement image (dotted line) of the cross-sectional area (C2) of FPL tendon (white arrow) at the midpoint of the first metacarpal bone

This study was approved by the Ethics Committee of our hospital. High-resolution ultrasound examinations were performed by experienced ultrasound physicians with a L5-12 transducer (iU-22 system, Philips Medical Systems, Andover, MA, USA). During the examination, the patient should keep quiet, sit quietly on the chair, put hands flat on the examination bed, palm up, and keep both wrist joints, MCP joints and interphalangeal joints straight. First, we placed the transducer horizontally at the level of the MCP joint of the tested thumb, scanned the FPL tendon horizontally and continuously, and carefully confirmed that the tendon and tendon sheath have no lesions. Then, at the MCP joint and the midpoint of the first metacarpal bone, we adjusted the inspection angle of transducer to make the acoustic beam perpendicular to the FPL tendon, so as to clearly display the FPL tendon. Finally, we selected the "Zoom" key to enlarge the FPL tendon and measured the cross-sectional area of the FPL tendon by automatic wrapping method ("Trace" key)to obtain the cross-sectional area of the MCP joint of FPL tendon (C1) and the midpoint of the first metacarpal of FPL tendon (C2) were analyzed (Fig. 1). There were three experienced ultrasound physicians participating in ultrasound examination. The first step was shared by three ultrasound physicians; the last two steps were completed by a doctor who completed the C1 and C2 measurements of all the people included in the study. The study involved two groups.

### First group: Healthy volunteers

Inclusion criteria: patients in the physical examination center of our hospital. Exclusion criteria: 1) Pregnancy or lactation; 2) Trauma history of FPL tendon; 3) History of tendon sheath diseases such as finger tendinosis, tendonitis, tenosynovitis, tendon sheath cyst, giant cell tumor of tendon sheath; 4) History of tuberculosis, skin, rheumatic immunity, metabolic or endocrine diseases, especially diabetes and gout;; 5) History of carpal tunnel syndrome; 6) Long-term use of steroids; 7) Workers or activists who need to repeatedly rub the FPL tendon sheath: such as carpenters, weightlifters, restaurant waiters, keyboard players, rock climbers or enthusiasts; 8) Left handedness; 9) Poor inspection compliance or tendon, tendon sheath or pulley disease found during the inspection. The following parameters were recorded during the evaluation: age, sex, C1 and C2 of both thumbs.

271 healthy volunteers were recruited in four different age groups (0- 6 year, 7–17 year, 18- 40 year and ≥ 41 year). The thumb of each volunteer's left and right hand was measured by ultrasound (a total of 542 thumbs). All samples were collected from March 2016 to October 2019. (Table 1).

**Table 1:** Clinical characteristics of healthy volunteers and patients with trigger thumb

Characteristic	Number (male: female)	Age (years)
Normal thumb	271(144: 127)	24.0(1- 80)
Group 1	64(39: 25)	4.5(1- 7)
Group 2	86(46: 40)	11.0(7- 17)
Group 3	62(30: 32)	30.3(19- 40)
Group 4	59(29: 30)	57.6(41- 80)
Trigger thumb	65(13: 52)	40.4(2- 65)
Group A	16(4: 12)	3.8 (2- 6)
Group B	49(9: 40)	52.4(41- 65)

Data are presented as mean (range) where applicable

Group1: 0 – 6 year, Group 2: 7–17 year, Group3:18- 40 year and Group4: ≥ 41 year

Group A: 0- 6 year) and Group B: ≥ 41 year

### Second group: Patients with trigger thumb

Inclusion criteria: orthopedic doctors in our hospital diagnosed patients with trigger thumb for the first time based on typical clinical symptoms and physical examination. Diagnosis is usually made on a clinical basis, in which a painful popping or clicking sound and locking of a thumb is elicited by flexion and extension of the trigger thumb. The most common presenting sign in patients with trigger thumb is tenderness or pain over the A1 pulley. Exclusion criteria: 1) Those who have a history of trigger finger disease and have been treated with drugs or surgery before; 2)pregnancy or lactation; 3) Trauma history of FPL tendon; 4) History of tendon sheath diseases such as finger tendinosis, tendonitis, tenosynovitis, tendon sheath cyst, giant cell tumor of tendon sheath; 5) History of tuberculosis, skin, rheumatic immunity, metabolic or endocrine diseases, especially diabetes and gout;; 6) History of carpal tunnel syndrome; 7) Long-term use of steroids; 8) Left handedness; 9) Poor inspection compliance or tendon, tendon sheath or pulley disease found during the inspection. The following parameters were recorded during the evaluation: age, sex, C1 and C2 of both thumbs, and marked the diseased side.

57 patients with a clinical diagnosis of trigger thumb in our hospital from March 2012 to March 2016 were collected, and a total of 65 affected thumbs were examined by ultrasonography. The 57 patients were divided into two groups according to age, Group A (0–6 years old) and Group B (≥ 41 years old). (Table 1) The data are from the patients collected in our previous trial [11]. In this trial, we regrouped them by age for statistical analysis.

### Statistical analysis

According to the normality of the data, the independent two-sample t-test, the paired t-test and one-way ANOVA were used to analyze data. Descriptive statistics included frequency (percentage) and mean ± standard deviation (Mean ± SD). To determine a diagnostic cut-off value of the ratio of C1 to C2 (C1/C2), we used the receiver operating characteristic (ROC) curve, calculated from the sonographic measurements of the patients. The significance level for all statistical analyses was set at 0.05.

## Results

### Sonographic measurements of healthy volunteers

**Fig. 2:**
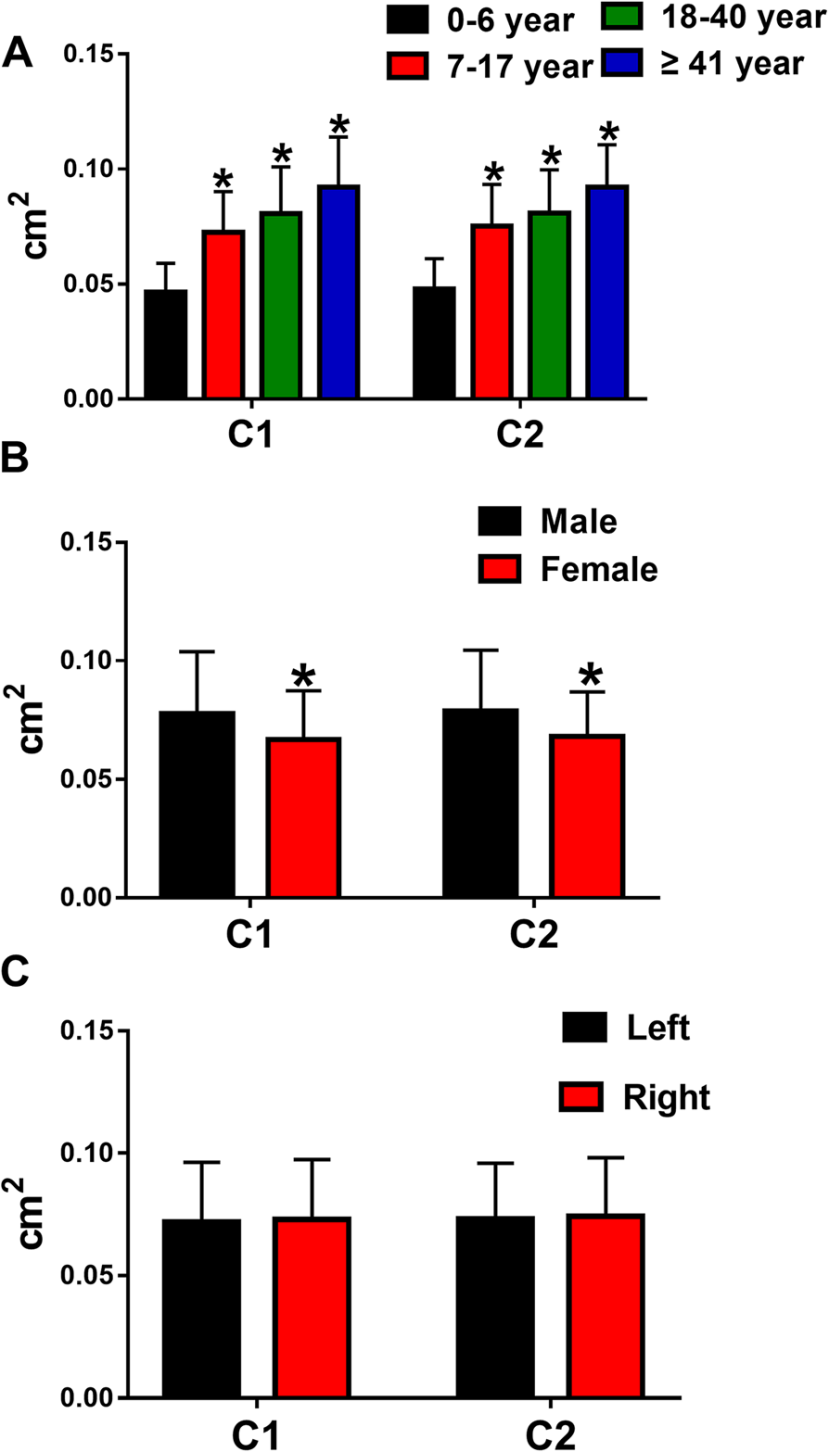
In the normal thumb, comparisons of the C1 and C2 in different ages, genders, and left and right hands. The asterisk indicates a significant difference (*P* < 0.05)

In the investigated healthy volunteers, the average of C1 and C2 were 0.072 ± 0.024 cm^2^, 0.074 ± 0.023 cm^2^. (Table 2) We found a significant difference in C1 and C2 in comparison to each different age. (Fig. 2A) The Group 4 (≥ 41 year) had the largest values, followed by Group 3 (18- 40 year), Group 2 (7- 17 year) and finally, the Group 1 (0- 6 year). (*P* < 0.05) There were differences in cross-sectional area between men and women. Males have a larger cross-sectional area than females. (*P* < 0.05) (Fig. 2B) In contrast, no difference in the cross-sectional area of ​​left and right hands. (*P* = 0.241, *P* = 0.132) (Fig. 2C).

**Fig. 3:**
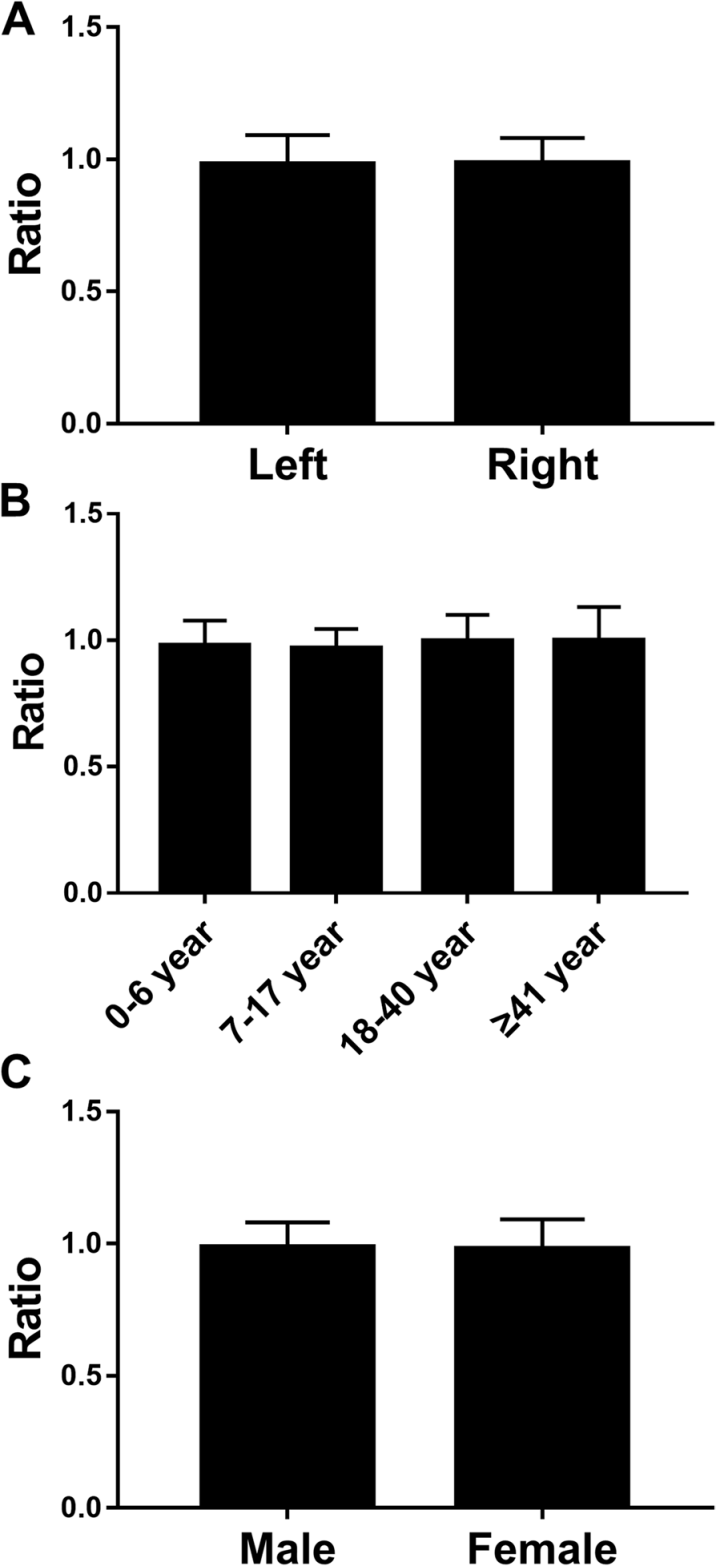
Comparisons of the C1/ C2 between gender, age, and left and right hands in the normal thumb

There is no difference between gender, age, and left and right hands in C1/ C2. (Fig. 3) The average of C1/ C2 was 0.983 ± 0.103. (Table 1).

### The relationship between clinical data and US measurements of patients

**Fig. 4:**
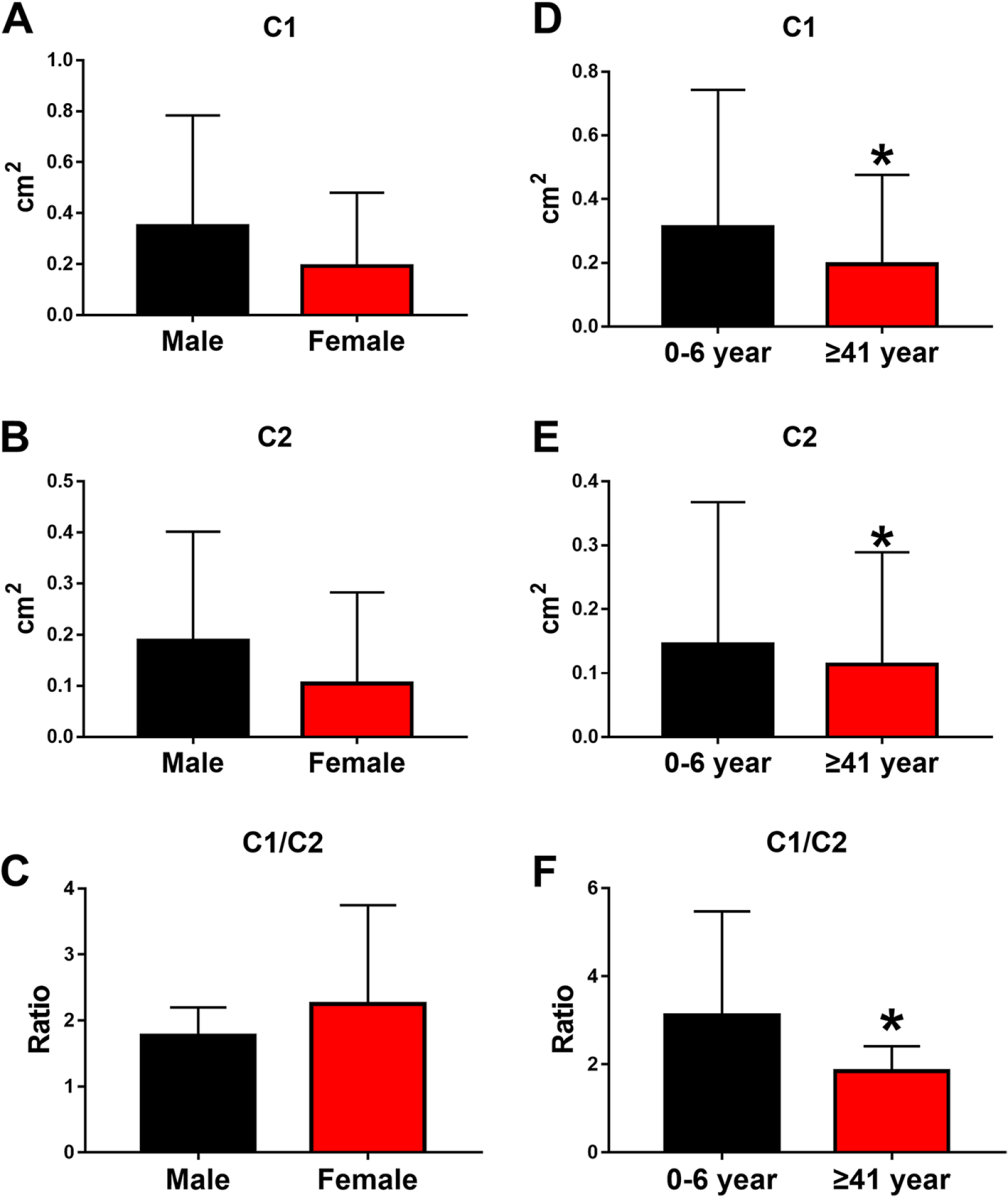
Comparisons of the C1, C2 and C1/ C2 in the trigger thumb

**Fig. 5:**
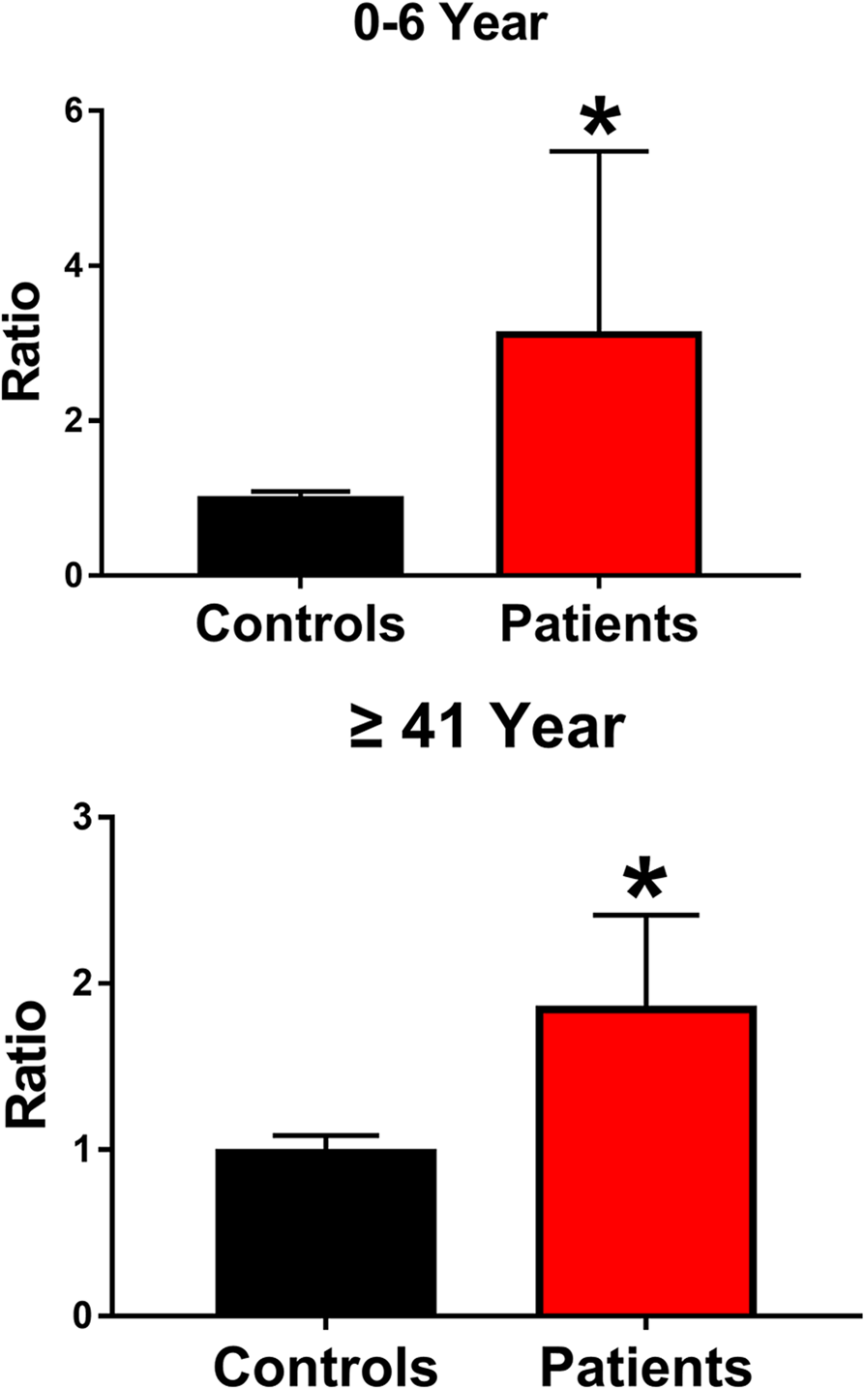
Comparisons of C1/ C2 in the normal thumb and trigger thumb. The asterisk indicates a significant difference (*P* < 0.05)

In patients with trigger thumb, the average of C1, C2 and C1/C2 were no statistically different between men and women (Fig. 4A- C). The C1, C2 and C1/ C2 of ​​group A (0- 6 year) is larger than that of group B (≥ 41 year). (*P* < 0.05) (Fig. 4D- F) The mean of C1/ C2 in different age groups were significantly larger than that of healthy people. (*P* < 0.05) (Fig. 5).

**Fig. 6:**
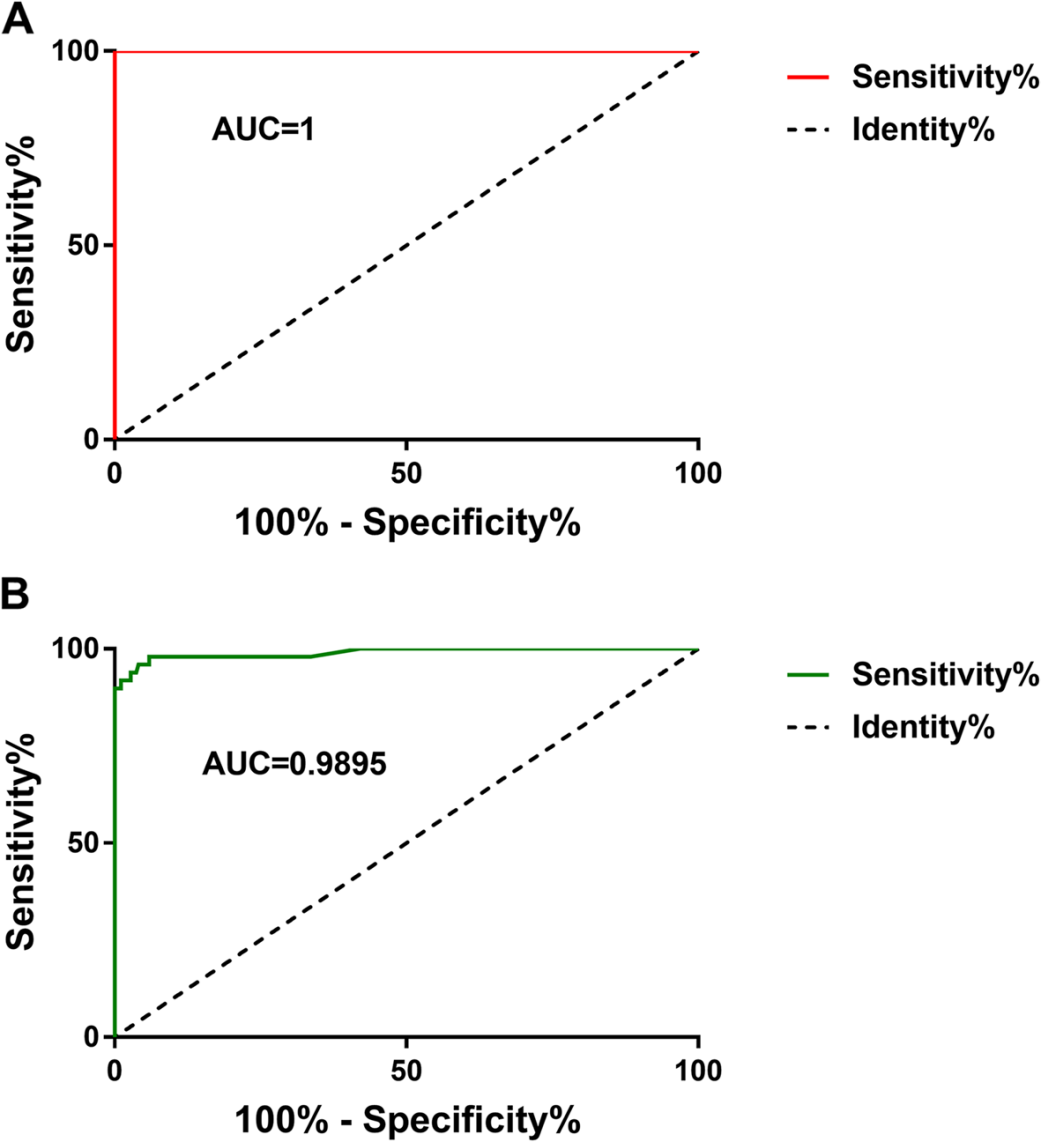
The measurements of the C1/ C2 in normal thumb are plotted against the measurements in trigger thumb. **A** The ROC areas under curve of the group A (0- 6 year) pulley was 1; **B** the ROC areas under curve of group B (≥ 41 year) was 0.9895. ROC = Receiver operating characteristic; AUC= the area under the ROC curve

Based on the ROC curve, we chose the diagnostic cut-off value for the C1/ C2 to be 1.362 and 1.153 in order to differ a trigger thumb from children and adults. The sensitivity and specificity of this cut-off value were shown to be 98.0% and 94.1% in adults; 100% and 100% in children. (Fig. 6).

**Table 2:** The sonographic information of measurement in normal thumb and trigger thumb

	C1 (cm^2^)	C2(cm^2^)	C1/C2
Normal thumb	0.072 ± 0.024	0.074 ± 0.023	0.983 ± 0.103
Group 1	0.046 ± 0.013	0.048 ± 0.013	0.978 ± 0.100
Group 2	0.073 ± 0.018	0.075 ± 0.018	0.967 ± 0.078
Group 3	0.081 ± 0.020	0.081 ± 0.019	0.996 ± 0.104
Group 4	0.092 ± 0.022	0.092 ± 0.018	1.000 ± 0.131
Trigger thumb	0.224 ± 0.324	0.122 ± 0.187	2.156 ± 1.362
Group A	0.312 ± 0.431	0.146 ± 0.222	3.112 ± 2.364
Group B	0.196 ± 0.281	0.114 ± 0.175	1.840 ± 0.081

In each box of the measurements, the mean value ± standard deviation is described in the upper box

C1 = the cross-sectional area of metacarpophalangeal (MCP) joint of flexor pollicis longus (FPL) tendon; C2 = the cross-sectional area of the midpoint of the first metacarpal of FPL tendon; C1/C2 = the ratio of C1 to C2

Group1: 0 – 6 year, Group 2: 7–17 year, Group3:18- 40 year and Group4: ≥ 41 year

Group A: 0- 6 year) and Group B: ≥ 41 year

## Discussion

The pathogenesis of trigger finger/thumb is not clear and is still under study. Many studies suggest that inflammation and age-related degeneration are very important in adult trigger finger/thumb. Kohler et al. found that the senescence of tendon stem cells in terms of cell size and functional adaptability may lead to tendon ageing and degeneration [12]. Therefore, the senescence of tendon stem cells caused their inability to maintain the tendon structure, thus interacting with mechanical factors, which may create a situation analogous to overuse. During this process, the balance between mechanical strain and tendon structure was disturbed, resulting in systemic inflammatory edema and mechanical (overuse) degradation of tendon. In this study, we found that C1 and C2 in the investigated healthy volunteers have significant differences between different ages. C1 and C2 showed an increasing trend with age, the Group 4 (≥ 41 year) had the largest values, which supported the above conclusion. At the same time, we also found that there was no significance between C1 and C2 of the left and right hands in the investigated healthy volunteers, which suggested that it was appropriate to use the normal contralateral thumb as the control in daily clinical work. Some studies have shown that trigger finger is more common in people with metabolic diseases (such as diabetes, hypothyroidism) [13]. In patients with diabetes, the lifetime incidence of trigger finger/thumb is 10%, while that of healthy people is 2.6% [1]. Therefore, we exclude patients with tuberculosis, skin, rheumatic immunity, metabolic or endocrine diseases.

In trigger finger/thumb, flexor tendons often become swollen and, on a transverse scan, their cross-sectional area is rounder than that of the adjacent unaffected tendons [14]. Our previous study found that the ratio (C1/C2) of the cross-sectional area of FPL tendon at the MCP joint (C1) to the cross-sectional area of the midpoint of the first metacarpal bone (C2) can be used to suggest trigger thumb [11]. Therefore, whether C1/C2 has a diagnostic cutoff value indicating trigger thumb, we further expanded the sample size for further exploration in this study. At present, studies have shown that diabetes has been identified as the main risk factor for trigger finger development, and it is also related to the duration of the disease [15]. Therefore, in the follow-up study, our team will consider analyzing the changes of C1/C2 in specific diseases and whether the diagnostic cutoff value we found is applicable.

In the ultrasound examination of the FPL tendon in normal people, we found that C1/ C2 was not affected by age, gender, and the left/ right hands, with a mean value of 0.983 ± 0.103. In patients with trigger thumb, we found that C1/ C2 of both adults and children is much higher than that of normal people. This was consistent with our previous test results [11], indicating that the repeatability and operability of this parameter were very good. Moreover, there was a significant difference in C1/ C2 between different age, the children’s C1/ C2 much larger than adults. This may be related to the different pathogenesis of adults and children. Various causes of adult’s trigger finger/thumb have been proposed, including repetitive finger movements or compressive forces at the A1 pulley and repetitive local trauma [1]. However, the pediatric trigger thumb was a common thumb disease in children. It was considered to be an acquired disease because of the size mismatch between the enlarged FPL tendon and the narrow oblique pulley [16]. Through software analysis of ROC curve, we defined a cutoff value to distinguish healthy thumb from diseased thumb for adults (1.153) and children (1.362), which supplied us with reliable, clear-cut features indicating the diagnosis trigger thumb. It was worth noting that our cut-off value was 100% sensitivity and specificity in diagnosing children’s trigger thumb. Our diagnostic method may play a more important role in the diagnosis of children's trigger thumb.

There are several limitations to our study. First, tendon abnormalities are inconstant findings. Kim et al*.* found blurring or irregularity of the tendon margins, tendon sheath effusion, and loss of the normal fibrillar echogenic pattern in 62%, 16%, and 14% of cases [6]. The C1/C2 may not be suitable for patients with normal flexor tendons. Second, the correlation between C1/C2 and prognosis is not involved in this study, which can be used as the next direction. Third, we did not analyze trigger finger patients with metabolic, immune, and other diseases, whose symptoms may be more serious. At the same time, we did not collect the severity and duration of patients. These data maybe very important for us to further analyze the correlation of multiple factors. The final limitation of the study arises from the unblinded sonography examiner, who knew clinically whether the involved digit was a trigger thumb or a healthy thumb. This is impossible to avoid since an experienced sonographer will diagnose this pathology sonographically as well as clinically.

## Conclusions

Trigger thumb is a pathologic condition of the digital pulleys and flexor tendons. There is no clear and reliable measurement parameter to represent the flexor tendon in trigger thumb. This study suggests the cross-sectional area ratio (C1/C2) of a specific part of the flexor pollicis longus tendon in healthy people obtained by high-frequency ultrasound is relatively stable, with an average value of 0.983 ± 0.103. The C1/ C2 of patients with trigger thumb is significantly increased. In order to distinguish between healthy and diseased, we defined a cutoff value for adults (1.153) and children (1.362), which supplied us with ultrasonic parameters for diagnosing trigger thumb.

## Data Availability

The datasets generated and/or analyzed during the current study are not publicly available, because they involve the personal information of healthy volunteers and patients, but can be available from the corresponding author on reasonable request.
